# Is There a Role for Genomics in the Management of Hypertension?

**DOI:** 10.3390/ijms18061131

**Published:** 2017-05-26

**Authors:** Jacopo Burrello, Silvia Monticone, Fabrizio Buffolo, Martina Tetti, Franco Veglio, Tracy A. Williams, Paolo Mulatero

**Affiliations:** 1 Division of Internal Medicine and Hypertension, Department of Medical Sciences, University of Turin, 10126 Turin, Italy; jacopo.burrello@gmail.com (J.B.); silvia.monticone@unito.it (S.M.); fabrizio.buffolo@gmail.com (F.B.); tetti.martina@gmail.com (M.T.); franco.veglio@unito.it (F.V.); tracyannwilliams48@gmail.com (T.A.W.); 2 Medizinische Klinik und Poliklinik IV, Klinikum der Universität München, Ludwig-Maximilians-Universität München, 80336 Munich, Germany

**Keywords:** monogenic hypertension, genomics, genome-wide association studies, epigenetics, pharmacogenomics

## Abstract

Hypertension (HTN) affects about 1 billion people worldwide and the lack of a single identifiable cause complicates its treatment. Blood pressure (BP) levels are influenced by environmental factors, but there is a strong genetic component. Linkage analysis has identified several genes involved in Mendelian forms of HTN and the associated pathophysiological mechanisms have been unravelled, leading to targeted therapies. The majority of these syndromes are due to gain-of-function or loss-of-functions mutations, resulting in an alteration of mineralocorticoid, glucocorticoid, or sympathetic pathways. The diagnosis of monogenic forms of HTN has limited practical implications on the population and a systematic genetic screening is not justifiable. Genome-wide linkage and association studies (GWAS) have identified single nucleotide polymorphisms (SNPs), which influence BP. Forty-three variants have been described with each SNP affecting systolic and diastolic BP by 1.0 and 0.5 mmHg, respectively. Taken together Mendelian inheritance and all GWAS-identified HTN-associated variants explain 2–3% of BP variance. Epigenetic modifications, such as DNA methylation, histone modification and non-coding RNAs, have become increasingly recognized as important players in BP regulation and may justify a further part of missing heritability. In this review, we will discuss how genetics and genomics may assist clinicians in managing patients with HTN.

## 1. Introduction

Hypertension (HTN) is the most frequent modifiable risk factor for cardiovascular disease that affects about 1 billion people worldwide [1,2]. Notwithstanding the major advances in understanding the pathophysiology of HTN and the introduction of new treatment options, HTN remains a major public health problem with a high socio-economic and health burden. Essential HTN is a multifactorial disease and the lack of a single identifiable cause complicates the treatment of this condition.

Blood pressure (BP) levels are affected by modifiable factors such as salt, alcohol consumption, obesity, physical activity and chronic stress. However, twin and family studies have demonstrated that 30–50% of the individual risk comes from genetic factors [3,4] and a family history of HTN increases the risk of developing HTN by four times [5,6]. Over the last few decades a wealth of studies was designed to understand the genetic basis of HTN and the associated molecular alterations. Three different approaches have been used.

The identification of genes involved in Mendelian forms of HTN by linkage analysis and next generation sequencing, with description of rare variants with a major impact on BP levels. This approach may identify the pathophysiological mechanism of a specific disorder and lead to a targeted therapy [7,8].

An approach based on families, twins and adopted children, or population studies using genome-wide linkage and association studies (GWAS). The latter can identify single nucleotide polymorphisms (SNPs), which influence the risk of developing high BP [8]. GWAS has also been applied to pharmacogenomic studies to identify genetic variants that regulate pharmacokinetics and pharmacodynamics of anti-hypertensive drugs [9].

More recently the study of epigenetic modifications has aroused growing interest; DNA methylation, histone modification and non-coding RNAs have become recognized as important players in several pathophysiological processes, including BP regulation [10].

Genetics is the study of the basis of heredity: how phenotypic features are transmitted from one generation to the next and how a specific gene causes a particular disease, whereas genomics is the study of the entire genome. In this review we discuss how genetics and genomics may assist clinicians in managing patients with HTN. For this purpose, Pubmed database, Medline, Cochrane Library and Biomed Central were searched for articles related to the key words “hypertension” (combined with “genetics”, “genomics”, “pharmacogenomics” and “epigenetics”) and “monogenic hypertension”. We selected the most relevant manuscripts pertinent to our review design, which comprised monogenic forms of hypertension, large-scale genome-wide associations studies, epigenetic modifications in hypertensive patients and pharmacogenomics.

## 2. Monogenic Forms of Hypertension

Over the last century, an increasing number of syndromes associated with high BP that are caused by single rare gene mutations have been identified [11].

The discovery of genes responsible for monogenic forms of HTN identified the kidney and adrenal glands as important players in the regulation of BP levels [12]. The majority of these syndromes are due to gain-of-function or loss-of-function mutations, which result in an alteration of mineralocorticoid, glucocorticoid, or sympathetic pathways (see Table 1).

Among the syndromes that affect the mineralocorticoid pathway, familial hyperaldosteronism is the most frequent. Glucocorticoid-Remediable Aldosteronism (GRA), or familial hyperaldosteronism type 1 (FH-1) results from an unequal crossing over between *CYP11B1* (encoding for 11 β-hydroxylase) and *CYP11B2* (encoding for aldosterone synthase), leading to ACTH-dependent aldosterone secretion, HTN, hypokalemia, low renin and high aldosterone levels. Low-dose glucocorticoids suppress aldosterone production and normalize BP and potassium levels [13].

For familial hyperaldosteronism type 2 (FH-2) the causative gene has not yet been identified. FH-2 is indistinguishable from sporadic primary aldosteronism (PA) except for the presence of more members affected by PA within the same family. The diagnosis of this condition is made after exclusion of other familial forms of PA [14].

Familial hyperaldosteronism type 3 (FH-3) is caused by mutations in *KCNJ5*, which encodes the inwardly rectifying potassium channel Kir3.4. Described mutations are located near or within the selectivity filter and are responsible for loss of ion selectivity, resulting in Na^+^ entry, membrane cell depolarization, opening of voltage-dependent calcium channels, increase in calcium intracellular concentration and aldosterone production [15]. Patients display severe hypokalemia, difficult-to-treat HTN and bilateral adrenal hyperplasia. Bilateral adrenalectomy is necessary in most cases.

Recently, a fourth type of familial hyperaldosteronism (FH-4) has been described [16]. FH-4 is caused by a germline gain-of-function mutation of *CACNA1H*, encoding for a T-type calcium channel. CACNA1H constitutive activation leads to calcium entry and increased aldosterone production [17].

Of note, somatic mutations in genes encoding ion pumps and channels (*KCNJ5*, *ATP1A1*, *ATP2B3*, *CACNA1D*) in adrenal cells have been identified in more than 50% of patients with sporadic APA [18,19]. Germline mutations in *CACNA1D* have been described in two patients with unexplained PA and complex neurological disorders (seizures and functional neurological abnormalities, resembling cerebral palsy) [20]. This syndrome was called PASNA (Primary Aldosteronism, Seizures and Neurologic Abnormalities). Patients with PASNA are not able to transmit the mutation to their offspring because of the severe neurological impairment.

Another Mendelian form of low-renin HTN is the Apparent Mineralocorticoid Excess (AME) syndrome. Cortisol has a strong agonist activity on mineralocorticoid receptor (MR) and is present in a 100× higher concentration in bloodstream. HSD11B2 (type 2, 11β-hydroxysteroid dehydrogenase), converting cortisol in cortisone, prevents its binding to MR. The loss-of-function mutation of *HSD11B2* leads to cortisol-dependent activation of the MR resulting in sodium retention, hypokalemia, metabolic alkalosis, suppressed renin and aldosterone levels and increased cortisol/cortisone ratio [21,22]. An acquired deficiency of this enzyme is determined by excessive liquorice intake (glycyrrihizic acid from liquorice inhibit HSD11B2).

**Figure 1:**
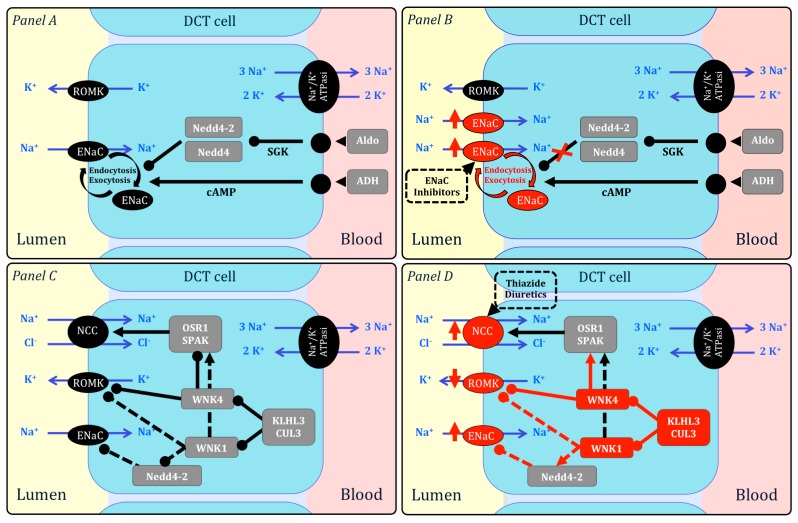
Gordon and Liddle syndromes. **Panel A**—Epithelial Na^+^ Channel (ENaC) is expressed in the distal convoluted tubule (DCT) at the apical membrane, where it allows Na^+^ in the lumen to enter the cell. At the baso-lateral membrane, Na^+^ is pumped outwards by Na^+^-K^+^ ATPase. ENaC membrane expression is regulated through membrane trafficking. Aldosterone (Aldo), vasopressin (ADH), Nedd4 and Nedd4-2 regulate membrane endocytosis and exocytosis, through cAMP-dependent pathways and aldosterone/serum and glucocorticoid-regulated kinase (SGK). **Panel B**—ENaC gain-of-function mutations determine resistance to Nedd-mediated ubiquitination with ENaC over-expression. ENaC inhibitors (such as amiloride) block ENaC and normalize BP. **Panel C**—Na^+^-Cl^−^ co-transporter (NCC), potassium channel (ROMK) and ENaC are responsible for Na^+^ and K^+^ homeostasis in DCT. Different intracellular factors regulate the activity of these transporters: WNK1 and WNK4 (kinases which inhibit NCC and ROMK), KLHL3 and CUL3 (ubiquitin ligases which mediate WNK kinases degradation). **Panel D**—The net effect of gain-of-functions mutations in WNK1 and loss-of-function mutations in WNK4, KLHL3 and CUL3 is the excessive activation of NCC and ENaC and the inhibition of ROMK, with increased reabsorption of sodium and reduced excretion of potassium. Thiazide diuretics block NCC and normalize BP. Arrows indicate up-regulation whereas lines ending in closed circles indicate down-regulation. Fine lines indicate established pathways, whereas dotted lines indicate pathways observed in in vitro models. The effects of mutations are indicated in red. Modified from Pathare 2013 [27] and Snyder 2002 [29].

Gordon’s syndrome, also known as type 2 pseudo-hypoaldosteronism or familial hyperkaliemic hypertension is characterized by HTN, hyperkalemia and hyperchloraemic metabolic acidosis [23] (see Figure 1). The diagnosis is mainly clinical with subsequent identification of the causal mutation, that however, is not obtained in all cases, indicating that as yet unidentified genes are associated with this condition [24]. To date, mutations of 4 genes have been described: mutations in WNK1 and WNK4 kinases [25], and more recently KLHL3 and CUL3 mutations [26]. The net effect of gain-of-function mutations in WNK1 and loss-of-function mutations in WNK4, KLHL3 and CUL3 is the excessive activation of sodium-chloride co-transporter (NCC) and epithelial sodium channel (ENaC) and the inhibition of the potassium channel ROMK, with increased reabsorption of sodium and reduced excretion of potassium [27]. The identification of molecular mechanisms underlying the pathology allows a targeted therapy with thiazide diuretics, which inhibit NCC, revert hyperkalaemia and normalize BP. Recently, *GILZ* (glucocorticoid induced leucine zipper protein) has been demonstrated to modulate renal potassium homeostasis; GILZ-knockout mice had hyperkalemia due to hyperstimulation of NCC, representing a reliable model of Gordon syndrome (even if mice had normal BP values) [28].

Liddle’s syndrome leads to hypokalemia, suppressed renin and aldosterone levels and a severe increase of BP, often resistant to anti-hypertensive agents, during childhood (see Figure 1). The disease is determined by gain-of-function mutations in the β- or γ- subunit of ENaC, with a consequent aldosterone-independent sodium retention. The mutations result in the inability to remove ENaC from the cell surface [29,30,31]. The treatment with ENaC inhibitors, such as amiloride, is highly effective in lowering BP and normalizing potassium levels.

Congenital adrenal hyperplasia (CAH) syndromes are a group of diseases caused by mutations in enzymes involved in glucocorticoid synthesis. In particular mutations in *CYP11B1* and *CYP17A1* (encoding for 11β-hydroxylase and 17α-hydroxylase, respectively) are associated with early onset HTN and hypokalemia. In these patients, ACTH is increased due to the absence of cortisol negative feedback resulting in adrenal hyperplasia and accumulation of cortisol precursors with mineralocorticoid activity (such as 11-deoxycorticosterone, DOC), which leads to HTN and hypokalemic alkalosis. Virilisation in affected females due to excessive androgen synthesis is also present in patients with *CYP11B1* deficiency [32] and pseudo-hermaphroditism in affected males with *CYP17A1* deficiency due to a deficit of androgen synthesis [33]. A CYP11B1 deficiency phenotype can also result from recombination between *CYP11B2* and *CYP11B1* genes thereby producing a hybrid *CYP11B2/B1* gene [34].

Hypertension with brachydactyly Type E is associated with a missense mutation of the *PDE3A* gene, encoding for phosphodiesterase 3A. This syndrome is characterized by severe salt-independent but age-dependent HTN and short phalanges and metacarpals [35,36]. The mutation increases protein kinase A-mediated PDE3A phosphorylation in vitro, which results in enhanced cell proliferation and contraction in mesenchymal stem cell-derived vascular smooth muscle cells. The mechanism underlying HTN development is not fully elucidated, but authors suggest that these alterations may produce vascular hypertrophy and increase of peripheral vascular resistances [37].

A form of HTN that is exacerbated by pregnancy is caused by a Ser810Leu mutation in the MR. The mutation results in constitutive activation of the receptor and is proposed to impair MR specificity such that some steroid hormone antagonists (such as progesterone and cortisone) act as agonists to the mutated receptor [38,39]. Affected women display severe HTN during pregnancy, with elevated progesterone levels; delivery improves HTN but there is no specific treatment for men and non-pregnant women. Spironolactone increases BP by activating the MR [38,39].

Among syndromes that affect sympathetic pathways about 30% of pheochromocytoma and paragangliomas are associated to germline mutations. The described mutations affect the equilibrium between oncogenes and onco-suppressor genes. In particular, mutations of *VHL*, *SDHA*, *SDHB*, *SDHC*, *SDHD* and *SDHAF2* alter the pathway related to hypoxia cellular responses mediated by hypoxia inducible factors, whereas mutations of RET and NF1 are associated to a constitutive activation of RAS/RAF/MEK and PI3KT/AKT/mTOR signalling [40]. Patients harbouring *VHL*, *RET or NF1* mutations show syndromic diseases, such as von Hippel-Lindau syndrome (retinal, cerebellar and spinal hemangioblastoma, renal cell carcinoma, pancreatic tumours and pheochromocytoma), type IIA multiple endocrine neoplasia (medullary thyroid carcinoma, parathyroid adenomas and pheochromocytoma) and type 1 neurofibromatosis (skin pigmentation, neurofibromas, brain tumours, and pheochromocytoma) [41]. Because of the impact on the diagnosis, prognosis and the possibility of early detection of the disease in relatives, the systematic targeted genetic screening should be considered in clinical practice for patients with a diagnosis of pheochromocytoma or paragangliomas.

These are some examples in which the description of a rare genetic mutation has enabled the understanding of disease pathogenesis and in some cases the identification of a molecular pharmacologically-targetable pathway. However the diagnosis of these syndromes has limited practical implications on the population, even in cases of a systematic genetic screening of all young hypertensives, which is not justifiable unless in the presence of features that suggest syndromic diseases.

## 3. Large-Scale Genome-Wide Associations Studies

The study of monogenic forms of HTN improved the knowledge of physiological mechanisms regulating BP. Nevertheless, Mendelian disorders are rare and do not explain the genetic component in patients with essential HTN. Indeed, most human genome variability is determined by SNPs. SNPs are structural variations of a single nucleotide, which occur in a specific position of the genome [42].

The large-scale identification of these polymorphisms has been made possible by linkage and association mapping [43]. Genetic linkage is the tendency of close alleles on a chromosome to be inherited together. Linkage analysis is based on this trend and identifies genetic regions of interest by mapping the genome of families with rare syndromes. This method has been highly successful for the identification of monogenic disorders, but it has also been applied in patients with essential HTN. The BRIGHT study supported the linkage of a locus on chromosome 5q13 with essential HTN susceptibility [44], whereas two other studies demonstrated a linkage with multiple loci on chromosome 1q [45,46].

Association mapping is a technique of mapping expression quantitative trait loci (eQTL), which link phenotypes and genotypes of a population, taking advantage of linkage disequilibrium [47]. Association mapping studies are better suited for complex genetic traits such as HTN but require a higher number of patients compared to linkage analysis and the extensive knowledge of all SNPs within the genome of the organism of interest [48].

Recent technological advances have opened the door to genome-wide association studies (GWAS). GWAS methodology is based on the determination of the statistical association between each SNP and the pressure phenotype through a linear regression analysis [12]. Pressure phenotype is influenced by environmental factors, including anti-hypertensive medications. In GWAS, BP levels are adjusted for age, sex and BMI; subsequently by convention, +15/+10 mmHg are added respectively to systolic blood pressure (SBP) and to diastolic blood pressure (DBP) in the presence of pharmacological therapy with at least one anti-hypertensive drug [49].

The two (rarely three or more) alleles for each SNP have different prevalence in the population; the least frequent allele is defined as the minor allele. The minor allele frequency (MAF) is inversely proportional to the total number of SNPs in the population [50].

Therefore, the statistical power of GWAS is proportional to sample size, MAF and each SNP-effect on SBP/DBP and inversely proportional to the number of independent tests performed (which is equal to the number of SNPs analysed). Therefore, SNPs with an allele frequency less than 5% have a too low predictive power, whereas more frequent variants may represent attractive targets for multifactorial diseases because of the expected stronger impact [51].

The first attempt to identify variants associated with HTN by GWAS was performed in 2007 on 2000 patients with HTN, but failed to identify any association probably because of the small sample size (GWAS by the Wellcome Trust Case Control Consortium) [52]. Later studies with larger sample sizes were subsequently performed: in 2011 the International Consortium for Blood Pressure GWAS (ICBP) completed the analysis of over 200,000 subjects [49], identifying 29 SNPs associated with HTN. Using candidate gene focused arrays, the described variants increased to 43 [53] but the effect size for each SNP was demonstrated to be about 1 mmHg for SBP and 0.5 mmHg for DBP (see Table 2) [12]. A risk score combining the effects of each variation was built with all the identified SNPs: the resulting score proved to be predictive of HTN if applied to different populations, but explained only a small part of the phenotypic variance among patients. Furthermore, it was also associated with the risk of stroke, coronary artery disease and left ventricular hypertrophy, but not with renal failure, indicating that the relationship between HTN and kidney function is more complex than expected [49].

Finally, only a minority of all the 43 described variants were near a gene known to be associated with BP levels [12]. The remaining SNPs were located in regions of the genome which were not thought to be related to HTN and were thus proposed as future sequencing targets to identify new biological pathways associated with pathophysiological alterations and potentially new therapeutic targets.

Taken together all GWAS-identified HTN-associated variants only explain 2–3% of BP variance [8,49], whereas the expected heritability varied between 30% and 50% [3,4]. However, studying a specific cohort under a specific environment overestimates the expected heritability and it is also likely that many more SNPs remain to be discovered [7].

## 4. Epigenetic Modifications in Hypertensive Patients

Epigenetics includes all heritable changes in gene expression regulation, which do not involve mutations in the DNA sequence [54]. Epigenetic modifications can be determined by many factors such as environmental influences during foetal life and childhood, exposure to chemical agents, aging, diet, medications and others [55]. Research in the field of epigenetic modifications provides insights on how the regulation of BP levels cannot be explained just by Mendelian inheritance [10,56] and could also justify a further part of the missing heritability, unexplained by GWAS. The processes that may cause epigenetic modifications of DNA include methylation, post-translational histone modification and mechanisms based on small non-coding RNAs [10] (see Table 3).

DNA methylation is the transfer of a methyl group from *S*-adenosyl-l-methionine to the 5′-position of a cytosine ring, to form 5-methyl-cytosine (5mC). This occurs in specific genomic regions called CpGs islands, composed of a series of dinucleotides, guanine and cytosine. Of note, CpG islands are often located in promoter regions [10]. DNA methylation down-regulates gene transcription resulting in gene silencing [55]. In patients with HTN the total number of 5mC is decreased with increasing HTN severity [57]. Furthermore, *HSD11B2* gene promoter methylation is associated with HTN onset [58], as well as hypomethylation of genes of the renin-angiotensin-aldosterone (RAA) system or *NKCC1* (Na-K-Cl co-transporter 1) gene respectively affect the response to ACE (angiotensin-converting enzyme) inhibitors and diuretics [59,60].

Histones package DNA into structural units called nucleosomes. Post-translational DNA modifications include histone acetylation, or less frequently methylation and phosphorylation [70]. Histone acetylation promotes gene transcription, whereas deacetylation produces the opposite effect [71]. Acetylation or deacetylation of eNOS (endothelial nitric oxide synthase), RAA system genes, NKCC1, NCC or WNK4 were demonstrated to affect vascular tone, renal hydro-electrolyte homeostasis and therefore BP [63,64,65,66].

Finally, small non-coding RNAs are also implicated in epigenetic modifications. In particular microRNAs (miRNA/miR) may down-regulate genes by binding the corresponding mRNA, resulting in the degradation of the latter and repression of translation [72]. miRNA with a possible role in BP regulation are listed in Table 3 [59,67,68,73].

Through these mechanisms the environment could influence genotype in each individual and these findings complicate the field of HTN genetics and genomics, adding another factor able to modify the phenotypic expression of patients with HTN [10].

## 5. Pharmacogenomics

Theoretically genomics can also be applied to predict drug response. The goal of pharmacogenomics is to identify genetic variants associated with a better response to a pharmacological class over another, in the attempt to administer “the right dose of the right drug to the right patient” [74]. In Table 4 we report some significant studies published on how a patient's genotype can affect pharmaco-kinetics or -dynamics of a given drug.

An example of this type of study is the SOPHIA study: 372 patients with HTN treated in monotherapy with losartan were included in the analysis. Through a whole-genome approach, the authors identified a significant association between 4 SNPs on *CAMK1D* gene and BP response. *CAMK1D* encodes for a calcium-dependent kinase that modulates the transcription of factors NURR1, ARF1, ATF2 and CREB, involved in aldosterone production in the adrenal *zona glomerulosa* [75].

Pharmacogenomics may also be applied to the identification of new therapeutic targets in patients with HTN. GWAS on Milan hypertensive rats and humans revealed the *ADD1* gene (encoding for α-adducin), as an eQTL associated to a significant increase in BP [76,77]. Adducin is a cytoskeletal protein consisting of two subunits: a α-subunit associated to either a β- or a γ-subunit. Mutated α-adducin stimulates the Na^+^/K^+^ ATPase in tubular renal cells, increasing Na^+^ reabsorption. Ouabain, a hormone released by hypothalamus and adrenal glands, is able to modulate Na^+^/K^+^ ATPase activity and natriuresis. Rostafuroxin antagonizes ouabain and lowers BP. Plasma ouabain levels are directly correlated with the number of copies of the mutated α-adducin gene [78]. The OASIS-HT [79] and the PEARL (ongoing) randomized controlled trials investigated the BP response to rostafuroxin through a pharmacogenomic approach.

Another example is uromodulin (*UMOD*) genes. In 2010, its minor allele was demonstrated to be associated with a lower risk of HTN [80] and reduced urinary uromodulin excretion [80]. *UMOD* encodes for a protein expressed in the kidney, exclusively in the thick ascending limb of the loop of Henle [81]. *UMOD* knockout models in vivo had lower SBP and were resistant to salt-induced changes of BP [82], whereas *UMOD* overexpression produced an increase in UMOD renal excretion and BP levels [83]. Furthermore, furosemide significantly increased natriuresis and decreased BP in the latter model compared to wild type controls [83]. These findings suggest that patients with increased urinary *UMOD* excretion may have an enhanced response to loop diuretics and that the uromodulin pathway may represent a new target for future drug development [81].

Although studies are promising with possible practical applications, these results should be interpreted with caution because their reproducibility was poor, suggesting that the observed associations may not be effective or that they are applicable only within specific sub-populations [84].

## 6. Conclusions

Genetic and genomics studies have provided major insights into understanding the pathogenesis of HTN. Identification of Mendelian syndromes allows an effective targeted therapy, however, they are relatively rare diseases with limited economic-health implications. On the other hand, essential HTN is very frequent but clinical applications of GWAS are undefined. The challenge lies in moving from the identification of SNPs associated with hypertension to the description of new pharmacologically-targetable causal molecular mechanisms. An example is the discovery of UMOD by GWAS studies and the subsequent description of a new potentially targetable pathway. Considering the complexity of BP regulation, only a minority of all pharmacologically targetable pathways are known. It is possible that further technological innovations will provide tools able to unequivocally define the genetic architecture of HTN and provide new therapeutic options for hypertensive patients. A future perspective could be the definition of scores on HTN-related SNPs to predict which patients will develop HTN and to which therapy they will respond better.

## Figures and Tables

**Table 1:** Hypertension (HTN)-related monogenic syndromes.

Monogenic Syndrome	Inheritance	Gene	Locus	Phenotype	Therapeutic Indications
Pheochromocytomas/Paragangliomas	Autosomal dominant	*SDHA* *SDHB* *SDHC* *SDHD* *SDHAF2* *TMEM127* *MAX*	5p15.3 1p36.13 1q23.3 11q23.1 11q12.2 2q11.2 14q23.3	Paragangliomas or pheochromocytomas.	Surgery/α adrenergic blockers
von Hippel–Lindau syndrome	Autosomal dominant	*VHL*	3p25.3	Retinal, cerebellar and spinal hemangioblastoma, renal cell carcinoma, pheochromocytomas, pancreatic tumours.	Surgery/α adrenergic blockers (for pheochromocytoma)
Multiple endocrine neoplasia, type 2A	Autosomal dominant	*RET*	10q11.2	Medullary thyroid carcinoma, parathyroid adenomas, pheochromocytoma.	Surgery/α adrenergic blockers (for pheochromocytoma)
Neurofibromatosis type 1	Autosomal dominant	*NF1*	17q11.2	Skin pigmentation, skin neurofibromas and brain tumours. Pheochromocytoma.	Surgery/α adrenergic blockers (for pheochromocytoma)
GRA–familial hyperaldosteronism type 1	Autosomal dominant	*CYP11B1 CYP11B2*	8q24.3	Familial form of PA	Glucocorticoids
Familial hyperaldosteronism type 2	Autosomal dominant	N.A.	7p22.3-7p22.1	Familial form of PA	Mineralocorticoid receptor antagonist/unilateral adrenalectomy (for APA)
Familial hyperaldosteronism type 3	Autosomal dominant	*KCNJ5*	8q24.3	Severe form of PA with bilateral adrenal hyperplasia	Bilateral adrenalectomy in drug-resistant patients
Familial hyperaldosteronism type 4	Autosomal dominant	*CACNA1H*	16p13.3	Familial form of PA	Mineralocorticoid receptor antagonist
PASNA syndrome	N.A.	*CACNA1D*	3p21.3	PA and complex neurological disorders (seizures and functional neurological abnormalities, resembling cerebral palsy).	N.A.
Sporadic APA	N.A.	*KCNJ5* *ATP1A1* *ATP2B3* *CACNA1D*	11q24.3 1p31.1 Xq28 3p21.3	Sporadic forms of PA.	Adrenalectomy
Pseudohypoaldosteronism, type 2 (Gordon’s syndrome)	Autosomal dominant (*dominant/recessive)	*WNK1* *WNK4* *CUL3* *KLHL3* *	12p12.3 17q21.2 2q36.2 5q31.2	HyperK^+^ hyperCl^−^ metabolic acidosis. Low PRA and low-normal AC.	Thiazide diuretics
Apparent mineralocorticoid excess (AME) Syndrome	Autosomal recessive	*HSD11B2*	16q22.1	Hypokalemia. Low PRA and AC. Increased cortisol/cortisone ratio.	Mineralocorticoid receptor antagonist
Liddle’s syndrome	Autosomal dominant	*SCNN1B*, *SCNN1G*	16p12.2	ENaC constitutive activation. Hypokalemia. Low PRA and AC.	ENaC blockers (amiloride, triamterene)
11β-hydroxylase deficiency	Autosomal recessive	*CYP11B1*	8q24.3	Virilisation, short stature. Low PRA and AC. HypoK^+^ alkalosis.	Glucocorticoids to inhibit ACTH-driven adrenal hyperpasia
17α-hydroxylase deficiency	Autosomal recessive	*CYP17A1*	10q24.3	HypoK^+^ alkalosis. Absent sexual maturation. Androgen deficiency.	Glucocorticoids to inhibit ACTH-driven adrenal hyperpasia
Hypertension with brachydactyly Type E	Autosomal dominant	*PDE3A*	12p12.3 12p12.1	Brachydactyly, short phalanges and metacarpals.	N.A.
Hypertension exacerbated by pregnancy	Autosomal dominant	*NR3C2*	4q31.23	Early onset hypertension exacerbated during pregnancy.	N.A.

GRA, glucocorticoid remediable aldosteronism. PA, primary aldosteronism. N.A., not available. PASNA, primary aldosteronism, seizures and neurologic abnormalities. APA, aldosterone producing adenoma. HyperK^+^, hyperkalemic. HyperCl^−^, hyperchloraemic. PRA, plasma renin activity. AC, aldosterone concentration. HypoK^+^, hypokalemic. ENaC, epithelial sodium channel. Modified from Padmanabhan et al. [7].

**Table 2:** Genetic variants associated with hypertension.

SNPs	Nearest Gene(s)	Position	Encoded Protein Function
rs880315	*CASZ*	1p36.22	Zinc finger transcription factor that acts as tumour suppressor.
rs4846049	*MTHFR(3′)-NPPB*	1p36.22	MTHFR catalyse the conversion of 5,10-MTH in 5-MTH and it is involved in homocysteine metabolism. NPPB encodes for the B natriuretic peptide.
rs17367504	*MTHFR(5′)-NPPB*		
rs17030613	*ST7L-CAPZA1*	1p13.2	ST7L is a tumour suppressor factor. CAPZA1 regulates growth of actin filaments.
rs2932538	*MOV10*	1p13.2	A component of the RISC complex RNA helicase.
rs2004776	*AGT*	1q42.2	Pre-angiotensinogen.
rs16849225	*FIGN-GRB14*	2q24.3	FIGN regulates microtubules synthesis. GRB4 is a growth factor receptor-binding protein, which interacts with insulin receptors and insulin-like growth factors.
rs13082711	*SLC4A7*	3p24.1	Sodium bicarbonate co-transporter in neuronal cells, involved in visual and auditory transmission.
rs3774372	*ULK4*	3p22.1	Serine/Threonine kinase involved in neurite branching and elongation and neuronal migration.
rs319690	*MAP4*	3p21.31	Promotion of microtubule assembly.
rs419076	*MECOM*	3q26.2	Transcriptional regulator and oncoprotein involved in apoptosis, hematopoiesis, cell differentiation and proliferation.
rs1458038	*FGF5*	4q21.21	Fibroblast growth factor 5, involved in embryonic development, cell growth, morphogenesis, tissue repair, tumour growth and invasion.
rs13107325	*SLC39A8*	4q24	Mitochondrial cellular import of zinc, involved in inflammation.
rs6825911	*ENPEP*	4q25	Glutamyl aminopeptidase; associated with renal neoplasm.
rs13139571	*GUCY1A3-1B3*	4q32.1	Guanylate cyclase 1 soluble subunit α, involved in nitric oxide pathway transduction.
rs1173771	*NPR3-C5orf23*	5p13.3	Natriuretic peptide receptor 3, responsible for clearing natriuretic peptides through endocytosis of the receptor.
rs11953630	*EBF1*	5q33.3	Early B-cell factor 1, associated with central obesity, B-lymphocytes differentiation and Hodgkin lymphoma.
rs1799945	*HFE*	6p22.2	Hemochromatosis protein; regulation of iron absorption.
rs805303	*BAT2-BAT5*	6p21.33	Genes cluster localized near genes for TNF α and β, involved in inflammatory process and associated with insulin dependent diabetes and rheumatoid arthritis.
rs17477177	*PIK3CG*	7q22.3	A catalytic subunit of PI3K, involved in the immune response.
rs3918226	*NOS3*	7q36.1	Endothelial nitric oxide synthase.
rs2898290	*BLK-GATA4*	8p23.1	BLK is a tyrosine kinase involved in cell proliferation and differentiation. GATA4 is a zinc finger transcription factor involved in embryogenesis and myocardial differentiation and function.
rs1799998	*CYP11B2*	8q24.3	Aldosterone synthase.
rs4373814	*CACNB2(5′)*	10p12.33	Member of a voltage-gated calcium channel superfamily, associated with Brugada and Lambert-Eaton myasthenic syndrome.
rs1813353	*CACNB2(3′)*		
rs4590817	*C10orf107*	10q21.2	Chromosome 10 open reading frame 107. Unknown function.
rs932764	*PLCE1*	10q23.33	Phospholipase involved in Ras pathway, associated with early onset nephrotic syndrome.
rs11191548	*CYP17A1-NT5C2*	10q24.32	CYP17A1 is the 17α hydroxylase, involved in the steroidogenic pathway; mutated in congenital adrenal hyperplasia. NT5C2 is a hydrolase involved in purine nucleotides metabolism.
rs1801253	*ADRB1*	10q25.3	Adrenoreceptor β1, which mediate physiological effects of epinephrine and norepinephrine.
rs7129220	*ADM*	11p15.4	Pre-hormone cleaved in adrenomedullin and pro-adrenomedullin, which act as vasodilator, hormone secretion regulators and angiogenesis promoters.
rs381815	*PLEKHA7*	11p15.2	Pleckstrin homology domain containing A7 associated with breast carcinomas and glaucoma.
rs633185	*FLJ32810-TMEM133*	11q22.1	FLJ32810 is a regulator of vascular tone. TMEM133 is a transmembrane protein; unknown function.
rs17249754	*ATP2B1*	12q21.33	Calcium ATPase with critical role in intracellular Ca^2+^ homeostasis
rs3184504	*SH2B3*	12q24.12	Signalling activities by growth factor and cytokine receptors; associated with celiac disease and insulin-dependent diabetes.
rs11066280	*ALDH2*	12q24.12	Mitochondrial aldehyde dehydrogenase 2, involved in the oxidative pathway of alcohol metabolism.
rs10850411	*TBX5-TBX3*	12q24.21	T-box protein family encoding for transcriptional factors regulating heart and limbs developmental processes.
rs1378942	*CYP1A1-ULK3*	15q24.1	CYP1A1 is a mono-oxygenases involved in drug catabolism and synthesis of cholesterol, steroid and other lipids. ULK3 is a serine/threonine kinase; unknown function.
rs2521501	*FURIN-FES*	15q26.1	FURIN is a protease, involved in the catabolism of PTH, TGFβ1 and other growth factors. FES is a tyrosine kinase, involved in hematopoiesis and cytokine receptor signalling.
rs13333226	*UMOD*	16p12.3	Regulation of renal sodium handling. See text.
rs17608766	*GOSR2*	17q21.32	Trafficking membrane protein.
rs12940887	*ZNF652*	17q21.32	Zinc finger protein, associated with breast and prostate cancer.
rs1327235	*JAG1*	20p12.2	Hematopoiesis regulation through notch 1 signalling.
rs6015450	*GNAS-EDN3*	20q13.32	GNAS is a G-protein that activates adenylyl cyclase with a wide variety of cellular responses. EDN3 encode for endothelin 3, implicated also in neural crest-derived cell lineages differentiation.

The table reports the 43 SNPs associated with blood pressure and the nearest gene (when there were two flanking genes, we reported both). Only a minority of the reported SNPs are near a gene known to be related to BP. Modified from Ehret et al. [12].

**Table 3:** Epigenetic modifications in hypertension.

Epigenetic Modification	Findings/Effectors	Reference
**DNA methylation**	Amount of 5mC in DNA inversely proportional to BP levels	Smolarek et al. [57]
	*HSD11B2* promoter methylation associated with HTN onset at young age	Friso et al. [58]
	RAA system gene hypomethylation associated with HTN in offspring, BP regulation and ACE inhibitor response.	Goyal et al. [59] Pei et al. [61] Rivière et al. [62]
	Hypomethylation of *SIC2A2* associated with NKCC1 overexpression, Na^+^ reabsorption and HTN.	Lee et al. [60]
**Histones acetylation/deacetylation**	Acetylation/deacetylation of endothelial cells nucleosomes regulate eNOS expression	Fish et al. [63]
	Nucleosomes modifications regulate ACE transcription	Lee et al. [64]
	Nucleosomes modifications regulate NKCC1 transcription and sodium renal reabsorption	Cho et al. [65]
	WNK4 down-regulation determines histones acetylation and NCC overexpression.	Mu et al. [66]
**Non coding RNA**	miR-27a and -27b down-regulate ACE1 mRNA	Goyal et al. [59]
	miR-155 down-regulate AGTR1 mRNA	Cheng et al. [67]
	miR-181a and -663 down-regulate renin mRNA and are under-expressed in HTN-patients	Marquez et al. [68]
	miR-425 down-regulate *NNPA*, leading to ANP under-production and salt overload	Arora et al. [69]

5mC, 5-methil-cytosine. BP, blood pressure. HSD11B2, hydroxysteroid 11-β dehydrogenase 2. HTN, hypertension. RAA, renin-angiotensin-aldosterone system. ACE, angiotensin converting enzyme. NKCC1, Na-K-Cl co-transporter 1. eNOS, endothelial nitric oxide synthase. NCC, NaCl co-transporter. miR, microRNA. AGTR1, angiotensin II type 1 receptor. ANP, Atrial natriuretic peptide. Modified from Wise et al. [10].

**Table 4:** Main genetic associations with response to anti-hypertensive drugs.

Genetic Association	Effect	Reference
*NEDD4L*, *PRKCA*, *GNAS-EDN3*, *TET2, CSMD1*, *HSD3B1*	Enhanced response to thiazide diuretics	Dahlberg et al. [85] Duarte et al. [86] Chittani et al. [87] Salvi et al. [88]
*ADRB1*	Enhanced response to metoprolol	Ganesh et al. [89]
*ADRB1*	Enhanced response to carvedilol	Mialet-Perez et al. [90]
*ADRB1*	Greater mortality in patients treated with verapamil than with atenolol	Karlsson et al. [91]
*CYP2D6*	Decrease in metoprolol clearance	Rau et al. [92]
*GRK4*	Enhanced response to atenolol	Bhatnagar et al. [93]
*SIGLEC12*, *A1BG*, *F5*	Higher risk of treatment-related adverse CV outcomes in patients treated with CCB	Hannila et al. [94]
*CAMK1D*	Enhanced response to losartan	Frau et al. [75]
*ADD1*	Enhanced response to rostafuroxin	Staessen et al. [79]

The table describes the effects of SNPs in genes indicated in the first column. CV, cardiovascular. CCB, calcium channel blockers. Modified from Cooper-DeHoff et al. [9].

## Abbreviations

Hypertension (HTN), Blood Pressure (BP), Genome-Wide Association Studies (GWAS), Single Nucleotide Polymorphisms (SNPs), Glucocorticoid Remediable Aldosteronism (GRA), Familial Hyperaldosteronism (FH), Primary Aldosteronism (PA), Primary Aldosteronism Seizures and Neurologic Abnormalities (PASNA), Apparent Mineralocorticoid Excess (AME), Mineralocorticoid Receptor (MR), 11β-Hydroxysteroid Dehydrogenase type 2 (HSD11B2), Sodium-Chloride co-transporter (NCC), Epithelial Sodium Channel (ENaC), Congenital Adrenal Hyperplasia (CAH), expression Quantitative Trait Loci (eQTL), Systolic Blood Pressure (SBP), Diastolic Blood Pressure (DBP), Minor Allele Frequency (MAF), Renin-Angiotensin-Aldosterone (RAA) system, Na-K-Cl Co-transporter 1 (NKCC1), Angiotensin-Converting Enzyme (ACE), endothelial Nitric Oxide Synthase (eNOS), microRNA (miRNA/miR), Uromodulin (UMOD).
